# Pragmatic Neuroethics: Lived Experiences as a Source of Moral Knowledge

**DOI:** 10.1017/S0963180118000105

**Published:** 2018-10

**Authors:** GABRIELA PAVARINI, ILINA SINGH

**Keywords:** neuroethics, morality, young people, pragmatism, moral behavior

## Abstract

In this article, we present a pragmatic approach to neuroethics, referring back to John Dewey and his articulation of the “common good” and its discovery through systematic methods. Pragmatic neuroethics bridges philosophy and social sciences and, at a very basic level, considers that ethics is not dissociable from lived experiences and everyday moral choices. We reflect on the integration between empirical methods and normative questions, using as our platform recent bioethical and neuropsychological research into moral cognition, action, and experience. Finally, we present the protocol of a study concerning teenagers’ morality in everyday life, discussing our epistemological choices as an example of a pragmatic approach in empirical ethics. We hope that this article conveys that even though the scope of neuroethics is broad, it is important not to move too far from the real life encounters that give rise to moral questions in the first place.

The study of morality…ceases to lust after timeless foundational principles in order to ask what actions and forms of social organization will best foster the flourishing of our biological and social natures.^1^

Neuroscience is traditionally committed to a descriptive understanding of brain, mind, and behavior, and conventionally grounded in empirical approaches. Ethics, on the other hand, commits itself to the complex normative question of how a good life should be lived. Unlike the science of the brain, ethics has traditionally relied on reason and argument as key methodological tools. Neuroethics, then, is a marriage of apparently mismatched partners. However, as in many marriages, each partner brings something to the table, making up what the other lacks in knowledge or skill. This article is an attempt to demonstrate the value of an integration of the normative and the empirical in neuroethics, through the framework of a pragmatic approach.

## The Pragmatic Approach in Neuroethics

The pragmatic approach has been variously interpreted, but it is perhaps at its core a “protest” against principlism and “foundationalism,” or the idea that knowledge can be grounded in a priori methods of inquiry (such as an appeal to abstract duties and obligations). A key disagreement within pragmatism is about the nature of “the good”; this is most clearly illustrated by the debate between the philosophers Richard Rorty^2,3^ and Hilary Putnam.^4,5^ Where Rorty defends a position of moral relativism that some associate with postmodernism, Putnam, following John Dewey and William James, insists that a “common good” can be discovered, under a precondition of democracy. Glenn McGee^6^ resolves the problem of the nature of morality by appealing to a “philosophic naturalism” that also refers back to John Dewey’s^7^ formulation of the *bios* as the foundational architecture of an organism’s potential for flourishing. A pragmatic account of philosophic naturalism makes the following claims:

• The “spectator” theory of knowledge cannot lead to correct understanding
• The knower and the known are part of nature, and dynamically intertwined
• One cannot confront moral problems as separate from daily experiences; the context of moral decisionmaking is crucial
• Notions of the right and the good can and will change over time; moral truth is not absolute

Pragmatism is an appropriate empirical approach in neuroethics, in part because it accepts science as having legitimate claims and methods. From a pragmatic perspective there are “facts” about human beings that shape their moral capacities; for example, the evolution of sociality and language, and the structure of cognitive development. At the same time, however, the claims set out here underline important epistemological limitations: “facts” about humans and the natural world are never received independently of human observation and interaction. For John Dewey, the task of pragmatic philosophy is to identify the moral issues at stake in a particular context, and to ask what forms of social life foster human flourishing. Dewey believed that a “common good” is discoverable, but that it requires systematic methodological inquiry and that the methodological approach must bridge philosophy and the social sciences, as follows:

1) Identify a “felt difficulty”
2) Describe its location and definition
3) Suggest a possible solution
4) Develop by reasoning the consequences of the suggestion
5) Engage in further observation and experimentation leading to acceptance or rejection of the suggested solution

Importantly, for Dewey, moral thought can be expressed as hypotheses, which can be subjected to ongoing experimental testing that should confirm, deny, or refine those hypotheses. In the present article, we approach neuroethics from this pragmatic ethical perspective, dividing the discussion according to the two familiar divisions of neuroethics: the “neuroscience of ethics” and the “ethics of neuroscience.”^8^ The former refers to the study of psychobiological mechanisms for morally relevant phenomena, including moral judgments, social emotions, selfishness, and prosocial behavior,^9,10^ and the latter concerns the study of ethical implications of progress in neuroscience and neighboring disciplines.^11,12^

In the following sections, we examine both domains of neuroethics from a pragmatic ethical perspective, briefly outlining the experimental data on moral cognition and action, and the use of empirical ethics to investigate moral behavior and experiences related to neuroscience intervention and innovation. We examine how neuroscientific evidence may be relevant to normative analysis, and how the “ethics of neuroscience” may be approached from an empirical standpoint. Finally, we describe a study concerning teenagers’ moral experiences, elucidating our epistemological choices and methodological strategies as an example of a pragmatic approach in empirical ethics.

## The “Moral Brain” and its Relevance to Normative Analysis

Functional imaging and patient studies have enriched our understanding of human morality by unravelling networks of brain regions implicated in moral cognition.^13,14^ One remarkably consistent finding is that judgments of both moral violations and virtuous behavior engage brain areas involved in emotional processing.^15,16,17^ This suggests, as philosopher David Hume had long proposed, that the distinction between right and wrong cannot be made by reason alone. Another important finding in moral neuroscience is that morality is multidimensional: partially dissociated neural systems seem to underlie judgments of different moral transgressions, such as acts of intentional harm, sexual deviance, and dishonesty.^18,19,20^

Beyond moral thinking, a substantial body of psychological and neuroscientific literature has investigated moral *action,* including prosocial and antisocial behavior. These studies have described a series of genetic and environmental factors that predict moral virtue and wrongdoing. For example, there is evidence that early exposure to social adversity, including poverty, violent crime, and abusive parenting, is a key risk factor for antisocial behavior,^21,22,23,24^ and that certain genotypes (e.g., a functional polymorphism in the gene encoding neurotransmitter-metabolizing enzyme monoamine oxidase A) can moderate children’s sensitivity to social risk.^25,26^ There is also evidence that prosocial and affiliative tendencies can be predicted from genotypic variations (e.g., variations of the oxytocin receptor gene),^27,28^ as well as environmental factors such as supportive parenting and availability of prosocial role models.^29^

Are such neuroscientific findings a valid basis from which to infer normative conclusions? Without ignoring the distinction between facts and values, or the “is” of science and the “ought” of ethics,^30^ we believe that neuroscientific evidence does bear relevance to normative analysis. A clear-cut example is that of research on factors that promote or preclude moral behavior, which shapes practice in clinics, schools, and the justice system as well as government funding priorities. However, scientific research *alone* does not reveal what one “ought” to do. For example, empirical studies may provide a metric for calculating risk for violent behavior, and describe possible outcomes of intervention initiatives, but would not directly answer the question of whether it is right or wrong to assess risk or to intervene. Empirical studies can, however, help test arguments that have been put forward in support of or against normative decisions in a particular context. To take the example given, one argument against assessing individuals (and informing them about) biological risk is stigmatization. In response to this argument, empirical studies can seek evidence as to whether this concern is well founded, and describe the boundary conditions. This particular approach in neuroethics, which attempts to integrate the normative and the empirical, will be explored in greater detail in the following section.

Another possible contribution of neuroscientific research to normative analysis is to clarify where moral judgments come from and how they work. One interesting example concerns moral judgments of behaviors that potentially taint the purity of the body, such as cannibalism, incest, and bestiality. Exposure to these (hypothetical) scenarios was found to recruit unique brain areas that are not activated during other types of moral judgments.^31,32^ Moreover, the revulsion that participants report in reaction to these scenarios was found not to vary as a function of the harm involved^33,34^ or of the protagonists’ intentions;^35,36,37^ furthermore, initial judgments persevered in the face of contradicting evidence.^38^ Is disgust a valid and justifiable basis for moral condemnation? These studies do not *directly* address this matter. However, they prompt ethicists and lawmakers to consider the potential influence of this emotion in legal regulation of human practices that relate to the physical body, including surrogacy, prostitution, and organ trading.

Another scientific finding in the field of morality that bears relevance to normative analysis is that of the contextual dependence of moral cognition. Anecdotal and empirical research suggest considerable variability in moral judgments across time and place: what is a mere breach of convention for one group is a serious transgression for another.^39^ Cultural differences are also noticeable at a neurobiological level. For example, a recent study found that different areas of the brain were recruited by Chinese and American participants during the experience of moral emotions (e.g., admiration for someone’s virtue).^40^ Another study found that a specific polymorphism of the oxytocin receptor gene, which has been consistently implicated in socio-emotional sensitivity, only predicted support-seeking behavior in American contexts but not Asian contexts.^41^

These findings prompt us to reevaluate preconceptions of morality, and to accept that perhaps scientific studies of moral judgment and emotion can only provide us with variable conclusions. Concurrently, any academic endeavor that aims to address what is right and wrong cannot be unbiased, as it will be limited by academics’ own moral-cognitive architecture. Any attempts to find solutions for moral problems should, therefore, take these limitations, and the social context, very seriously. Across the globe, from schools to justice systems, from parenting to medical care, normative claims should be made with reference to what the context affords, and to culturally specific conceptions of the good and the good society.

## Ethics of Neuroscience and the Value of Empirical Approaches

A second—and not completely distinct—branch of neuroethics centers on ethical implications of advances in neuroscience to the individual and society as a whole. This subfield has been constructed mainly by ethicists, philosophers, law theoreticians, and social scientists. Several ethical issues have been voiced. One set of concerns, for example, surrounds neuropharmacological interventions to improve cognitive and emotional functions in healthy humans. Challenges range from the possible threats that these interventions pose to people’s authenticity, to questions of fairness and ethical implications for society at large.^42^ A second set of concerns derive from (the possibility of) using biomarkers to predict psychopathology and very early interventions to prevent cognitive and social difficulties.^43,44^ Ethical issues include the possibility of the early label becoming a self-fulfilling prophecy, and the question of whether such interventions impose a definition of authentic living and contravene a child’s “right to an open future.”

Another line of research centers on implications of advanced neuroscience to people’s self-concepts and attributions of intention and responsibility. For example, does neuroscience encourage a view of the self that is based in biology, and does it increase or reduce stigma attached to particular psychopathologies?^45^ Similarly, does accurate neuroprediction of human choices reduce people’s sense of agency and free will?^46,47^ These questions have important real-world implications for the ways in which we choose to reward or punish individuals for their actions. For example, would a “brain-disordered” individual be equally responsible, and deserving of equal punishment for a moral transgression, as a typically functioning person?^48^

These themes have been debated on both empirical and theoretical grounds. Even though the broader field of bioethics has traditionally drawn upon normative reasoning using tools and approaches from philosophy, the discipline has taken an “empirical turn.”^49,50^ Leading voices of this movement, for the most part sociologists, have stressed the need for contextualized ethical analysis, grounded in the experiences and attitudes of different stakeholders.^51.52,53^ These researchers advocated for the need of empirical research studies that are specifically designed to inform a particular bioethical debate, and whose results could in turn affect policy and practice within contextual boundaries.

This general advice has certainly been taken on board by neuroethicists, who are increasingly adopting empirical approaches. One example is that of research on the ethical issues associated with genotyping individuals for risk of Alzheimer’s disease. Considerable empirical efforts have been dedicated to addressing possible concerns that may influence the development of laws and regulations. For example, Robert Green et al.^54^ have looked at whether participants told about a risky genotype (vs. a control, nondisclosure group) experience distress and anxiety as a result. Others have examined laypeople’s views about taking such tests and their level of interest in doing so,^55,56,57^ and have gathered psychiatrists’ moral attitudes regarding the use of these tests and patient safeguards.^58^ These pieces of research are timely, given that direct-to-consumer saliva-based tests that allow individuals to independently ascertain their risk of developing a disease or disorder are already available in several countries.

How do normative claims arise from empirical data, and when is the evidence sufficient for making such claims? At present, there is no agreement among neuroethicists regarding how the empirical and the normative should be articulated.^59^ In a systematic review of empirical bioethics methodologies, Rachel Davies, Jonathan Ives, and Michael Dunn^60^ found striking variation in how much weight is placed on data versus theory, and in the way that researchers articulate normative conclusions. Using one approach, normative conclusions arrive before data collection finishes; that is, stakeholders and researchers engage in dialogue and reach a shared normative conclusion or solution to a particular problem. An alternative approach consists in collecting stakeholders’ perspectives, but analyzing data and drawing normative conclusions independently. It is very important that ethicists are aware of the different ways that normative justifications can arise from an empirical process in order to make methodological choices that are optimal for the research question at hand and the types of normative claims they wish to produce, and that align with their own theoretical approaches.

Because of its inherent interdisciplinarity, the field of neuroethics is also methodologically diverse. The lack of commitment to a common orientation or paradigm allows for a full range of methodological possibilities, from participant observation to qualitative interviews, from quantitative surveys to participatory action research. This lack of consistency should not, however, lessen methodological rigor and scientific quality. Again, it is essential that empirical neuroethicists have a solid and comprehensive understanding of research methods and that they are critical and reflexive about their methodological choices. When a research design is realized in this frame of mind, methodological flexibility within bioethics turns into an opportunity for generating novel methods that cross disciplinary boundaries.

Despite the methodological plurality, empirical neuroethicists share a common assumption: that ethics is rooted in the context in which it is lived and that ethical commitments are formed through lived encounters. This assumption has methodological implications: moral attitudes and intuitions that are collected during the research process are also contextually sensitive. In fact, even the wording and framing of hypothetical moral dilemmas presented to research participants (e.g., kill vs. save) can impact on their reported intuitions.^61^ Moral judgments are also subject to social influence;^62^ therefore, it matters whether participants are asked to complete the study alone or in a group setting. It also matters whether the group includes hierarchical relationships (e.g., physicians and patients) given that even momentary feelings of social power (or the lack thereof) can influence moral judgments.^63^ It is important that neuroethicists acknowledge that methodological choices are not independent of the target phenomenon, and that an explicit effort is taken to reflect on how one’s choices may have shaped the research findings and constrained their interpretation. This process may be better explored through an example of a real research study, which will be presented in the following section.

## Teenagers’ Moral Experiences in Everyday Life: An Empirical Ethics Study

To illustrate some of the aspects discussed in this article, we present a case study of empirical ethics research conducted as part of a Wellcome Trust-funded project entitled: “Becoming Good: Early Intervention and Moral Development in Child Psychiatry”. The study, *Digital Diaries: Young People’s Moral Experiences in Everyday Life*, investigates moral experiences of 12–18-year-olds using a digital diary methodology (i.e., participants reporting on moral experiences in real life settings using mobile devices). The study aims at identifying what young people consider to be “right” and “wrong” behavior in themselves and others, and what moral domains are relevant in their everyday lives (e.g., trust, loyalty, authenticity).

This study was designed partially in response to the overwhelming focus on children’s and teenagers’ character education in England over the past 10 years.^64^ It is now widely accepted that schools should contribute not only to the acquisition of skills and competences but also to morality and character development,^65,66,67^ and large government grants have been awarded to support projects that aim to enhance children’s moral traits, including generosity, respect, honesty, and self-control.^68^ There is strong hope that promoting these traits can positively impact the physical and financial health of the population, and reduce criminal offending.^69,70,71^ Despite such strong focus on character development, we found it surprising that the literature still lacked an understanding of young people’s *own* views vis-à-vis morality: what do they consider good and bad in their actions and those of others?

As advances in neuroscience and genetics progressively bring us closer to employing invasive, long-lasting interventions that may profoundly shape children’s moral and behavioral development at early stages,^48^ we and others find that there is little or no understanding of young people’s perspectives on such interventions. This situation contravenes the United Nations Convention for the Rights of the Child,^72^ which states that children have the right to express their own views in all matters that affect them. The moral problem that prompted our research was formulated in this lacuna in understanding: young people ought to be provided an opportunity, and enabled, to contribute to the design of interventions aimed at shaping moral character and intervening in “bad” behavior. Because of the lack of prior research in this area, the question driving our investigation is foundational: What are young people’s beliefs, attitudes, and values in response to the question, what does it mean to be “good”?

Drawing on the pragmatic approach, we generated several hypotheses to test in the study. We largely wanted the understanding of moral virtue to be generated from the data; therefore, our hypotheses are necessarily general. As we are also testing the feasibility of the method, we included this in our set of hypotheses as well:

1) Daily diary methodology will provide a reliable tool for data collection on young people’s everyday moral experiences
2) Online, phone-based data collection methodology will be accessible and interesting for young people involved in the research
3) Young people will be able to identify and to report multiple instances of morally good and bad behavior over the course of a week
4) Young people’s understanding of morally good and bad behavior will be moderated by immediate context (e.g., who is around them at the time) and by broader environment (e.g., their sociocultural position)
5) Young people’s reported moral behavior will vary across and within individuals

In what follows, we describe our protocol and the rationale behind some of our key methodological choices, as an illustrative case of the challenges involved in designing empirical ethics research.

We designed the study in such a way that data collection would be fully completed online through participants’ own mobile phones. Each participant received daily text messages for 4 consecutive days with a link to a short survey about morally relevant experiences. Once each day, they were asked to briefly describe (in free text form) anything that they had done over the past 24 hours that they thought was good or admirable, and anything they had done over the same period that they thought was bad or wrong. They were also asked to report on anything good or admirable and anything bad or wrong that they may have seen others doing toward themselves or third parties. Finally, they were asked to describe their thoughts and feelings in each situation. Participants were not given a definition of morality, or examples of “good” and “bad” behavior; they were asked to write honestly and openly and speak from their *own* perspective.

A few issues motivated us to perform this study online and using momentary ecological assessment. One is the dynamic and malleable nature of morality and high intra-individual variation. For example, there is evidence to suggest that when individuals perform what they consider to be a “good deed” they are more likely to subsequently engage in antisocial behavior without worrying about feeling or appearing selfish, a phenomenon named “moral self-licensing.”^73,74^ A cross-sectional, one-time assessment of moral attitudes would not only be less reliable, but would also prevent us from examining how potentially interesting moral dynamics unfold over time.

Diary methods and momentary longitudinal assessments have been used widely in psychological research as a method for understanding how thoughts, feelings, physiological states, and behavioral patterns unfold in ordinary life. It has been used to assess changes in well-being,^75^ mental health symptoms,^76,77^ physiological stress,^78^ and physical mobility,^79^ among many others. Despite its recent popularity, there has been only a handful of studies in which this method was adopted to assess morally relevant behavior.^80,81^

Our study adopts a specific type of momentary assessment that has been titled “Daily Reconstruction Method” (DRM).^82^ DRM prompts participants to report on experiences that happened within a time frame (in our case, the past 24 hours) instead of “in the moment” that the signal arrives. We considered that having one signal per day would not only reduce the burden of repeated measurements, but would also allow us to gather richer reports that covered full-fledged, uninterrupted moral experiences.

A second reason for adopting this methodology was that moral behavior tends to be sensitive to privacy. For example, psychological research has repeatedly suggested that individuals are reluctant to act unethically when being watched. In laboratory settings, participants were more likely to cheat when they thought the act would be completely private.^83,84^ Another study found that the frequency of littering at a British university cafe was reduced to half when subtle cues of being watched (i.e., posters displaying images of eyes) were present in the environment,^85^ compared with a control condition (i.e., posters featuring flowers). This research motivated us to adopt a highly private methodological tool: a survey to be completed on one’s mobile phone shortly after the relevant action, and in the absence of an experimenter (or other people) and to be submitted anonymously. We hoped that this would allow participants to report a greater number of misdeeds, and to be open about their intentions.

It is worth noting that we do not claim that our approach is free of bias. For example, morality may change just by virtue of reflecting on it. Indeed, experimental studies have suggested that reflecting on one’s moral qualities can activate participants’ moral identity and inspire value-consistent behavior.^86,87^ In one study, participants who were asked to write down “what they were grateful for” exhibited an increase in well-being over the course of a week.^88^ Similarly, participants may adjust their behavior simply by virtue of knowing that someone they know is taking part in the study (e.g., if participants are recruited through schools). As previous research suggests, individuals not only strive to maintain a positive moral image,^89,90^ but also compare their moral standing to that of others.^91^

With respect to the study’s goals, we did not design this study aiming to answer the question of what morality *is*, but we did hope to achieve an understanding of what is considered morally relevant by teenagers in different contexts. As we apply this study and methodology to different communities and groups, we hope to gather a rich understanding of how conceptions of good and bad depend on teenagers’ environment and life experiences. Therefore, an ecological analysis of teenagers’ responses with reference to their social context, age, history, and status is a key part of our data processing and interpretation, along with an analysis of the contextual limitations of the study itself, and what could have been achieved via alternative methods. Finally, we also hoped that this research study would help us to achieve a fuller understanding of teenagers as developing agents and citizens, and the ways in which they view and construct their moral lives.

## Concluding Remarks

We have reflected on the link between empirical methods and normative questions, using as our platform recent neuropsychological and bioethical research into moral cognition, action, and experience. We hope to have demonstrated (1) the normative value of a scientific understanding of the moral brain, and (2) the value of empirical approaches in the examination of related neuroethical dilemmas. We have argued that a pragmatic approach allows for integration of the normative and the empirical in neuroethics, a challenge that refers back to John Dewey, and his articulation on the “common good” and its discovery through a systematic experimental approach. This approach involves the acknowledgement that the research object (in this case, moral attitudes and decisions) is not independent of the observer, and that methodological choices can powerfully affect what is observed. The pragmatic approach also involves an acknowledgement that notions of right and wrong are dependent on historical and cultural contexts. A consideration of such contextual factors should be central to the empirical effort to understand moral decisionmaking and to address normative questions.

At a very basic level, the pragmatic approach considers that morality is not dissociable from lived experiences and everyday conduct. The case study we offer as illustration of a pragmatic approach argues that morality unfolds in everyday experiences: quarrelling with someone who has betrayed one’s trust, rescuing a friend who is in terrible danger, choosing to flee one’s home country to escape war. The scope of neuroethics is appropriately broad, but we should be careful not to move too far from the real life encounters that gave rise to moral questions in the first place. Our project also has a broader goal, to bring young people into neuroethics debates by developing systematic tools, in partnership with young people, that allow Deweyian hypothesis testing and iteration at scale. The study we have presented here represents a first step in a series of studies, in which we build ever more elegant and grounded models and tools for investigation of young people’s moral experiences and attitudes, as these relate to medical and neuroscience innovation and intervention.

## Notes

1. ArrasJ. Pragmatism in bioethics: Been there, done that PaulEF, MillerJr FD, PaulJ, eds. Bioethics. Cambridge: Cambridge University Press; 2002:29–58, at 38.

2. RortyR. Objectivity, Relativism, and Truth: Philosophical Papers, Volume 1. Cambridge: Cambridge University Press; 1991.

3. RortyR. Philosophy and Social Hope. London: Penguin Books; 1999.

4. PutnamH. Pragmatism: An Open Question. New York: Wiley-Blackwell; 1995.

5. PutnamH. The Many Faces of Realism. Chicago: Open Court; 1987.

6. McGeeG, ed. Pragmatic Bioethics. Cambridge, MA: MIT Press; 2003.

7. DeweyJ (HickmanL, AlexanderT, eds.). The Essential Dewey, 2 vols Bloomington: Indiana University Press; 1999.

8. RoskiesA. Neuroethics for the new millenium. Neuron 2002;35:21–3.1212360510.1016/s0896-6273(02)00763-8

9. CasebeerWD. Moral cognition and its neural constituents. Nature Reviews Neuroscience 2003;4:840–6.1452338310.1038/nrn1223

10. GreeneJ, HaidtJ. How (and where) does moral judgment work? Trends in Cognitive Sciences 2002;6(12):517–23.1247571210.1016/s1364-6613(02)02011-9

11. FarahMJ. Emerging ethical issues in neuroscience. Nature Neuroscience 2002;5(11):1123–9.1240400610.1038/nn1102-1123

12. LevyN. Neuroethics: Ethics and the sciences of the mind. Philosophy Compass 2009;4(1):69–81.

13. See note 10, Greene, Haidt 2002.

14. Sinnott-ArmstrongW, ed. Moral Psychology, Volume 3: The Neuroscience of Morality: Emotion, Brain Disorders, and Development. Cambridge, MA: MIT Press; 2008.

15. DamasioA. Looking for Spinoza. Orlando, FL: Harcourt; 2003.

16. GreeneJD, SommervilleRB, NystromLE, DarleyJM, CohenJD. An fMRI study of emotional engagement in moral judgment. Science 2001;293:2105–8.1155789510.1126/science.1062872

17. SanfeyAG, RillingJK, AronsonJA, NystromLE, CohenJD. The neural basis of economic decision-making in the ultimatum game. Science 2003;300(5626):1755–8.1280555110.1126/science.1082976

18. Sinnott-ArmstrongW, WheatleyT. Are moral judgments unified? Philosophical Psychology 2014;27(4):451–74.

19. LewisGJ, KanaiR, BatesTC, ReesG. Moral values are associated with individual differences in regional brain volume. Journal of Cognitive Neuroscience 2012;24(8):1657–63.2257145810.1162/jocn_a_00239PMC3383838

20. ParkinsonC, Sinnott-ArmstrongW, KoralusPE, MendeloviciA, McGeerV., WheatleyT. Is morality unified? Evidence that distinct neural systems underlie judgments of harm, dishonesty, and disgust. Journal of Cognitive Neuroscience 2011;23:3162–80.2145295110.1162/jocn_a_00017

21. BeaverKM, Eagle SchuttJ, BoutwellBB, RatchfordM, RobertsK, BarnesJC. Genetic and environmental influences on levels of self-control and delinquent peer affiliation: Results from a longitudinal sample of adolescent twins. Criminal Justice and Behavior 2009;36(1):41–60.

22. MoffittTE, CaspiA. Childhood predictors differentiate life-course persistent and adolescence-limited antisocial pathways among males and females. Development and Psychopathology. 2001;13(2):355–75.1139365110.1017/s0954579401002097

23. ScarpaA, OllendickTH. Community violence exposure in a young adult sample: III. Psychophysiology and victimization interact to affect risk for aggression. Journal of Community Psychology 2003;31(4):321–38.

24. WalliniusM, DelfinC, BillstedtE, NilssonT, AnckarsäterH, HofvanderB. Offenders in emerging adulthood: School maladjustment, childhood adversities, and prediction of aggressive antisocial behaviors. Law and Human Behavior. 2016;40(5):551–63.2724336010.1037/lhb0000202

25. CaspiA, McClayJ, MoffittTE, MillJ, MartinJ, CraigIW, et al Role of genotype in the cycle of violence in maltreated children. Science 2002;297:851–4.1216165810.1126/science.1072290

26. ByrdAL, ManuckSB. MAOA, childhood maltreatment, and antisocial behavior: Meta-analysis of a gene-environment interaction. Biological Psychiatry 2014;75(1):9–17.2378698310.1016/j.biopsych.2013.05.004PMC3858396

27. KoganA, SaslowLR, ImpettEA, OveisC, KeltnerD, SaturnSR. Thin-slicing study of the oxytocin receptor (OXTR) gene and the evaluation and expression of the prosocial disposition. Proceedings of the National Academy of Sciences 2011;108(48):19,189–92.10.1073/pnas.1112658108PMC322846822084107

28. TostH, KolachanaB, HakimiS, LemaitreH, VerchinskiBA, MattayVS, et al A common allele in the oxytocin receptor gene (OXTR) impacts prosocial temperament and human hypothalamic-limbic structure and function. Proceedings of the National Academy of Sciences 2010;107(31):13,936–41.10.1073/pnas.1003296107PMC292227820647384

29. HastingsPD, UtendaleWT, SullivanC. The socialization of prosocial behavior In: GrusecJE, HastingsPD, eds. Handbook of Socialization: Theory and Research. New York: Guilford Press; 2007:638–64.

30. For a detailed discussion see McMillanJ. Empirical bioethics and the fact/value distinction In: IvesJ, DunnM, CribbA, eds. Empirical Bioethics: Theoretical and Practical Perspectives. New York: Cambridge University Press; 2016:17–32.

31. BorgJS, LiebermanD, KiehlKA. Infection, incest, and iniquity: Investigating the neural correlates of disgust and morality. Journal of Cognitive Neuroscience 2008 20(9):1529–46.1834598210.1162/jocn.2008.20109PMC3969035

32. See note 20, Parkinson et al. 2011.

33. Giner-SorollaR, BossonJK, CaswellTA, HettingerVE. Emotions in sexual morality: Testing the separate elicitors of anger and disgust. Cognition and Emotion 2012;26(7):1208–22.2241421410.1080/02699931.2011.645278

34. GutierrezR, Giner-SorollaR. Anger, disgust, and presumption of harm as reactions to taboo-breaking behaviors. Emotion 2007;7(4):853.1803905410.1037/1528-3542.7.4.853

35. AstutiR, BlochM. The causal cognition of wrong doing: Incest, intentionality, and morality. Frontiers in Psychology 2015;18(6):136.10.3389/fpsyg.2015.00136PMC433230925741304

36. RussellPS, Giner-SorollaR. Moral anger, but not moral disgust, responds to intentionality. Emotion 2011;11(2):233.2150089210.1037/a0022598

37. YoungL, TsoiL. When mental states matter, when they don’t, and what that means for morality. Social and Personality Psychology Compass 2013;7(8):585–604.

38. RussellPS, Giner-SorollaR. Moral anger is more flexible than moral disgust. Social Psychological and Personality Science 2011;2(4):360–4.

39. For several examples, see PrinzJ. The Emotional Construction of Morals. New York: Oxford University Press; 2007.

40. Immordino-YangMH, YangXF, DamasioH. Correlations between social-emotional feelings and anterior insula activity are independent from visceral states but influenced by culture. Frontiers in Human Neuroscience 2014;16(8):728.10.3389/fnhum.2014.00728PMC416521525278862

41. KimHS, ShermanDK, SasakiJY, XuJ, ChuTQ, RyuC, et al Culture, distress, and oxytocin receptor polymorphism (OXTR) interact to influence emotional support seeking. Proceedings of the National Academy of Sciences 2010;107(36):15,717–21.10.1073/pnas.1010830107PMC293662320724662

42. SavulescuJ, MeulenR, KahaneG., eds.. Enhancing Human Capacities. Oxford: Wiley-Blackwell; 2011.

43. SinghI, RoseN. Biomarkers in Psychiatry. Nature 2009;460(7252):202–7.1958776110.1038/460202a

44. WalshP, ElsabbaghM, BoltonP, SinghI. In search of biomarkers for autism: Scientific, social and ethical challenges. Nature Reviews Neuroscience 2011;12:603–12.2193133510.1038/nrn3113

45. O’ConnorC, JoffeH. How has neuroscience affected lay understandings of personhood? A review of the evidence. Public Understanding of Science 2013;22(3):254–68.2383305310.1177/0963662513476812PMC4107825

46. NahmiasE, ShepardJ, ReuterS. It’s OK if ‘my brain made me do it’: People’s intuitions about free will and neuroscientific prediction. Cognition 2014;133(2):502–16.2519507710.1016/j.cognition.2014.07.009

47. RoseD, BuckwalterW, NicholsS. Neuroscientific prediction and the intrusion of intuitive metaphysics. Cognitive Science 2015;41(2):482–502.2645262910.1111/cogs.12310

48. For an interdisciplinary collection of perspectives on this topic, see SinghI, Sinnott-ArmstrongWP SavulescuJ., eds. Bioprediction, Biomarkers, and Bad Behavior: Scientific, Legal and Ethical Challenges. New York: Oxford University Press; 2013.

49. AshcroftRE. Constructing empirical bioethics: Foucauldian reflections on the empirical turn in bioethics research. Health Care Analysis 2003;11(1):3–13.1451030510.1023/A:1025329811812

50. BorryP, SchotsmansP, DierickxK. The birth of the empirical turn in bioethics. Bioethics 2005;19(1):49–71.1581297210.1111/j.1467-8519.2005.00424.x

51. ForliniC, RacineE, VollmannJ, SchildmannJ. How research on stakeholder perspectives can inform policy on cognitive enhancement. American Journal of Bioethics. 2013;13(7):41–3.10.1080/15265161.2013.79488223767439

52. Singh I. Evidence, epistemology and empirical bioethics. In: Ives 2016 (see note 30), at 17–32

53. StrechD, SchildmannJ. Why the “Appraisal of Guidelines for Research and Evaluation” instrument can and should further inform ethics policy work. American Journal of Bioethics 2012;12(11):25–7.10.1080/15265161.2012.71927223072676

54. GreenRC, RobertsJS, CupplesLA, RelkinNR, WhitehousePJ, BrownT, et al Disclosure of APOE genotype for risk of Alzheimer’s disease. New England Journal of Medicine 2009;361(3):245–54.1960582910.1056/NEJMoa0809578PMC2778270

55. HippsY, RobertsJS, FarrerLA, GreenRC. Differences between African Americans and Whites in their attitudes toward genetic testing for Alzheimer’s disease. Genetic Testing 2003;7(1):39–441282070110.1089/109065703321560921

56. RobertsJS, BarberM, BrownTM, CupplesLA, FarrerLA, LaRusseSA, et al Who seeks genetic susceptibility testing for Alzheimer’s disease? Findings from a multisite, randomized clinical trial. Genetics in Medicine 2004;6(4):197–203.1526620710.1097/01.gim.0000132688.55591.77

57. NeumannPJ, HammittJK, MuellerC, FillitHM, HillJ, TettehNA, et al Public attitudes about genetic testing for Alzheimer’s disease. Health Affairs 2001;20(5):252–64.1155871110.1377/hlthaff.20.5.252

58. HoopJG, RobertsLW, Green Hammond KA, Cox NJ. Psychiatrists’ attitudes regarding genetic testing and patient safeguards: A preliminary study. Genetic Testing. 2008;12(2):245–52.1845239510.1089/gte.2007.0097

59. For a discussion of different approaches, see MolewijkB, StiggelboutAM, OttenW, DupuisHM, KievitJ. Empirical data and moral theory. A plea for integrated empirical ethics. Medicine, Health Care and Philosophy 2004;7(1):55–69.10.1023/b:mhep.0000021848.75590.b015139255

60. DaviesR, IvesJ, DunnM. A systematic review of empirical bioethics methodologies. BMC Medical Ethics 2015;16(1):15.2588557510.1186/s12910-015-0010-3PMC4357052

61. PetrinovichL, O’NeillP. Influence of wording and framing effects on moral intuitions. Ethology and Sociobiology 1996;17:145–71.

62. Le FurgyWG., WoloshinGW. Immediate and long-term effects of experimentally induced social influence in the modification of adolescents’ moral judgments. Journal of Personality and Social Psychology 1969;12:104–10.578471110.1037/h0027563

63. McGeeJ.A. The power to judge: Social power influences moral judgment. The Jury Expert 2013; 25:1–7.

64. Department for Education. Developing character skills in schools: Summary report, 2017; available at https://www.gov.uk/government/uploads/system/uploads/attachment_data/file/634710/Developing_Character_skills-synthesis_report.pdf (last accessed 4 June 2018).

65. DeweyJ. Moral Principles in Education. Boston: Houghton Mifflin; 1909.

66. GoodmanJ F, LesnickH. The Moral Stake in Education: Contested Premises and Practices. New York: Longman; 2000.

67. NucciLP, KrettenauerT, NarváezD, eds. Handbook of Moral and Character Education. New York: Routledge; 2008.

68. Department for Education. Character education: Apply for 2015 grant funding. 2015; available at https://www.gov.uk/government/news/character-education-apply-for-2015-grant-funding (last accessed 4 June 2018).

69. DoyleO, HarmonCP, HeckmanJJ, TremblayRE. Investing in early human development: timing and economic efficiency. Economics and Human Biology. 2009;31;7(1):1–6.1921361710.1016/j.ehb.2009.01.002PMC2929559

70. KnudsenEI, HeckmanJJ, CameronJL, Shonkoff, JP. Economic, neurobiological, and behavior perspectives on building America’s future workforce. Proceedings of the National Academy of Sciences 2006; 103:10,155–62.10.1073/pnas.0600888103PMC150242716801553

71. MoffittTE, ArseneaultL, BelskyD, DicksonN, HancoxRJ, HarringtonH, et al A gradient of childhood self-control predicts health, wealth, and public safety. Proceedings of the National Academy of Sciences 2011;108(7):2693–8.10.1073/pnas.1010076108PMC304110221262822

72. United Nations General Assembly. Convention on the Rights of the Child, Treaty Series; 1989.

73. MoninB, MillerDT. Moral credentials and the expression of prejudice. Journal of Personality and Social Psychology 2001;81:33–43.11474723

74. MerrittAC, EffronDA, MoninB. Moral self-licensing: When being good frees us to be bad. Social and Personality Psychology Compass 2010;4(5):344–57.

75. EmmonsRA, McCulloughME. Counting blessings versus burdens: An experimental investigation of gratitude and subjective well-being in daily life. Journal of Personality and Social Psychology 2003;84:377–89.1258581110.1037//0022-3514.84.2.377

76. De VriesMW, CsikszentmihalyiM. The Experience of Psychopathology: Investigating Mental Disorders in Their Natural Settings. Cambridge: Cambridge University Press; 2006.

77. DelespaulP. Assessing schizophrenia in daily life: The experience sampling method. Maastricht: Maastricht University Press; 1995.

78. van HollandBJ, Frings-DresenMH, SluiterJK. Measuring short-term and long-term physiological stress effects by cortisol reactivity in saliva and hair. International Archives of Occupational and Environmental Health 2012;85(8):849–52.2218304810.1007/s00420-011-0727-3PMC3480579

79. LathiaN, SandstromGM, MascoloC, RentfrowPJ. Happier people live more active lives: Using smartphones to link happiness and physical activity. PLoS One 2017;12(1):e0160589.2805206910.1371/journal.pone.0160589PMC5213770

80. HofmannW, WisneskiDC, BrandtMJ, SkitkaLJ. Morality in everyday life. Science 2014;345(6202):1340–3.2521462610.1126/science.1251560

81. BollichKL, DorisJM, VazireS, RaisonCL, JacksonJJ, MehlMR. Eavesdropping on character: Assessing everyday moral behaviors. Journal of Research in Personality 2016;61:15–21.2711004410.1016/j.jrp.2015.12.003PMC4836053

82. KahnemanD, KruegerAB, SchkadeDA, SchwarzN, StoneAA. A survey method for characterizing daily life experience: The day reconstruction method. Science 2004;306(5702):1776–80.1557662010.1126/science.1103572

83. GinoF, AyalS, ArielyD. Contagion and differentiation in unethical behavior: The effect of one bad apple on the barrel. Psychological Science 2009;20(3):393–8.1925423610.1111/j.1467-9280.2009.02306.x

84. MazarN, AmirO, ArielyD. The dishonesty of honest people: A theory of self-concept maintenance. Journal of Marketing Research 2008;45(6):633–44.

85. Ernest-JonesM, NettleD, BatesonM. Effects of eye images on everyday cooperative behavior: a field experiment. Evolution and Human Behavior. 2011;32(3):172–8.

86. AquinoK, McFerranB, LavenM. Moral identity and the experience of moral elevation in response to acts of uncommon goodness. Journal of Personality and Social Psychology 2011;100(4):703.2144337510.1037/a0022540

87. SchnallS, RoperJ. Elevation puts moral values into action. Social Psychological and Personality Science. 2012;3(3):373–8.

88. See note 75, Emmons, McCullough 2003.

89. AquinoK, ReedAII. The self-importance of moral identity. Journal of Personality and Social Psychology 2002;83(6):1423.1250082210.1037//0022-3514.83.6.1423

90. SanitiosoR, KundaZ, FongGT. Motivated recruitment of autobiographical memories. Journal of Personality and Social Psychology 1990;59(2):229.221349210.1037//0022-3514.59.2.229

91. PavariniG. SchnallS. Is the glass of kindness half full or half empty? Positive and negative reactions to others’ expressions of virtue In: SarkissianH, WrightJC, eds. Advances in Experimental Moral Psychology. New York: Bloomsbury Academic; 2014:55–72.

